# Protocol and Preliminary Findings from the BrainPALS Study in Very Preterm Children: A Randomized Controlled Trial of a Digital Parenting Program

**DOI:** 10.3390/children13060774

**Published:** 2026-06-02

**Authors:** Dhawal Sanjay Bandrey, Christie S. Vieux, Haley M. Laughlin, Cara Price, Susan Landry, Johanna R. Bick, Dana M. DeMaster

**Affiliations:** 1 Department of Epidemiology, The University of Texas Health Science Center at Houston, Houston, TX 77030, USA; dhawalbandrey@gmail.com; 2 Children’s Learning Institute, The University of Texas Health Science Center at Houston, Houston, TX 77030, USA; cara.price@uth.tmc.edu (C.P.); susan.landry@uth.tmc.edu (S.L.); 3 Department of Cognitive Sciences, Rice University, Houston, TX 77005, USA; vieuxchristie@gmail.com; 4 Psychology Department, University of Houston, Houston, TX 77204, USA; haley.laughlin@times.uh.edu (H.M.L.); jrbick@uh.edu (J.R.B.)

**Keywords:** prematurity, self-regulation, executive function, emotion regulation, responsive parenting, neurodevelopment, randomized control trial

## Abstract

Background/Objectives: Children born very preterm (VPT; <33 weeks gestation) are at increased risk for atypical neurodevelopment and deficits in self-regulation, including executive function (EF) and emotion regulation (ER). Responsive parenting interventions, such as Play and Learning Strategies (PALS), improve caregiver behavior; however, their effects on neurodevelopmental outcomes in VPT populations remain limited. This study describes a randomized controlled trial evaluating a digital adaptation of PALS (ePALS). Methods: Families of toddlers aged 15–28 months born very preterm were randomized to either the ePALS intervention or an active control condition. Assessments were conducted at pre-test and post-test to evaluate caregiver responsiveness, child EF and ER, and neural development using behavioral tasks, EEG, and MRI. Results: To date, 84 families have been randomized (43 ePALS, 41 control). Preliminary findings indicate that parents in the ePALS condition demonstrate greater improvements in responsiveness compared to the control group. Intervention adherence rates are reported. Conclusions: These findings support the feasibility and preliminary efficacy of a digitally delivered parenting intervention for improving caregiver responsiveness in families of VPT toddlers. Ongoing analyses will evaluate long-term behavioral and neurodevelopmental outcomes.

## 1. Introduction

Prematurity (<37 weeks of gestation) is a significant public health problem, with preterm birth impacting 10% of births in the United States [1] and, specifically, 11.1% in Texas, where one in nine babies are born preterm [2], imposing considerable strain on healthcare systems and families. Preterm infants are typically classified into three categories based on gestational age: extreme preterm (<28 weeks; EPT), very preterm (28–32 weeks; VPT), and late preterm (32–36 weeks; LPT) [1], each associated with varying risks for medical and developmental complications [3].

Whereas survival rates of infants born at extreme and very preterm gestational ages have increased over the last two decades, early interventions to address the long-term neurodevelopmental consequences of prematurity have lagged behind. One critical consequence of prematurity is the infant’s immediate pain response [4]. An additional consequence of prematurity is demand on life-sustaining functions that maintain physiological homeostasis, such as breathing, nutrition, temperature regulation, and regulation of response to stimuli [5]. These demands for life-sustaining systems in the perinatal period precede the typical developmental timeframe, placing significant stress on an underdeveloped physiology, potentially a driving factor in atypical brain development. This atypical brain development for children born preterm is particularly notable in neurocircuitry associated with self-regulation [6,7,8]. Specifically, the prefrontal-limbic system, essential for controlling attention, emotion, and behavior [8] and associated areas. Neuroimaging studies have indicated modified connectivity and atypical maturation in these areas correlating with noted behavioral challenges in preterm newborns [9]. Children born preterm frequently exhibit motor delays [10], language problems, and deficits in self-control and emotional regulation [11]. Early intervention with responsive parenting programs [12] has become a viable approach to mitigate these long-term developmental problems. However, one challenge for the current clinical practice paradigm is the capacity to extend treatment from medical intervention primarily targeting NICU care to intervention targeting factors in the home environment with the potential to mitigate risk for later behavioral problems.

Emergent self-regulation skills in toddlers include emotion regulation, the ability to modulate negative emotions to achieve goals, and executive function, including sustained attention, inhibition, and working memory, which are impacted by prematurity-related delays, and these delays have been linked with enduring neurodevelopmental conditions, including ADHD and mood disorders [13]. Self-regulation is a key focus of home-based interventions, as the development of regulatory skills is often disrupted in preterm infants and can be influenced by the home rearing environment [14]. The U.S. Surgeon General has recognized parental stress as a significant public health concern [15], particularly among parents of preterm infants, who endure heightened stress of parenting a child at higher medical risk, and the interruption of typical attachment development resulting from extended NICU admissions. Whereas previous research shows that preventative early parenting interventions can significantly improve developmental outcomes related to self-regulation in high-risk infants [16], including promoting secure attachment with caregivers, social engagement, and reduced emotional distress among infants and children [17], supporting busy and under-resourced caregivers to sustain responsive caregiving during daily activities represents a significant challenge. From this perspective, it is important to consider the mechanisms through which parenting behavior shapes self-regulatory skills. The broad theoretical framework guiding this research posits that a caregiving environment rich in responsive and warm interactions helps regulate infant distress responses through parent–infant co-regulation, while also supporting healthy brain maturation [18]. Early childhood is characterized by rapid development of limbic systems involved in emotional reactivity, particularly the amygdala, which shows accelerated growth and heightened responsivity during the first four years of life. During this period, emotionally reactive signals are generated before the regulatory “stop gates” of the prefrontal cortex and anterior cingulate cortex are fully mature and capable of exerting top-down control. Given the prolonged development of the prefrontal cortex, children rely on caregiver responsiveness to co-regulate emotional and behavioral responses prior to achieving independent self-regulation.

Importantly, responsive parenting programs offer a research-to-practice, evidence-based approach to alleviate the increased stress levels of parents while providing an opportunity to practice responsive parenting behaviors. Responsive parenting programs that focus on meeting the parents’ goals for their interaction with their child, while including practice of responsive parenting skills during daily family activities, offer the most significant potential for sustained use by parents and are positioned to have the greatest impact on child outcomes. Research also shows that highly responsive parenting impacts normal brain development by fostering neuronal plasticity in the prefrontal-limbic system [19]. From this perspective, consistent warm and contingent interactions by caregivers offer a potential strategy to reduce neurodevelopmental consequences of preterm delivery, fostering more normative self-regulation and cognitive outcomes. Early interactions between parents and children are essential in developing the brain and behavior of toddlers, particularly those born very preterm [20]. Interventions that improve parental responsiveness may offer significant developmental benefits and early self-regulation and brain development for these children [21]. Considering these challenges, intervention programs like Play and Learning Strategies (PALS) give parents evidence-based tools to create sensitive and developmentally appropriate interactions [10]. Studies have shown that parental response to the PALS program results in favorable outcomes in cognitive development, executive functioning, and emotional regulation in preterm infants [10,22]. PALS was initially developed as an infant intervention (PALS I) and evaluated for effectiveness compared to an attention control condition involving equal home visits focused on infant development education. Results showed that mothers in the PALS I group significantly improved responsive behaviors—such that PALS-related changes in parental behavior positively affected infants’ cognitive and social development [10]. Specifically, maternal behaviors such as maintaining infant attention, providing rich verbal input, and offering contingent encouragement were linked to gains in infant language use and cooperation. These effects were observed in both term and very low birth weight (VLBW) infants, highlighting the tailored effectiveness of the response. PALS I was then adapted for use with parents of toddlers (PALS II) and was also found to be effective [10]. A unique feature of PALS is self-reflection, a tool rarely included in parent-focused interventions. Self-reflection allows parents to critique their own behaviors while watching a video of themselves interacting with their child. This is a powerful technique for parents to identify ways to use PALSs to meet their child’s needs better [23]. In older children, longitudinal observations indicate that parenting behavior that is high in psychological control negatively affects offspring’s decision-making abilities at the transition to adulthood. These more controlling and less responsive patterns of parenting, likely beginning early [24], such as PALS, can have enduring effects by reducing the likelihood of later behavioral and emotional difficulties. Consistent with this framework, emerging neuroimaging evidence indicates that children whose parents participate in structured parenting interventions exhibit increased brain connectivity in the prefrontal cortex, which is essential for self-regulation and decision-making functions [25,26].

This protocol paper describes the design and methodology of a randomized controlled trial (RCT) evaluating the effectiveness of the PALS parenting intervention, implemented with parents of toddlers born preterm. The broad scope of this RCT is to examine the extent to which a targeted early intervention improves caregiver responsiveness and, ultimately, supports more optimal developmental outcomes in this high-risk population. Available data collected and preprocessed to date is evaluated to test the impact of the PALS intervention on parent-responsive behaviors and child outcomes. The primary aim is to evaluate the feasibility of the PALS parenting intervention among caregivers of toddlers born preterm, operationalized as caregivers’ ability to enroll in and complete the 10-session intervention compared with a control group. The secondary aims are to assess the efficacy of the PALS. Specifically, the first secondary aim is to determine whether participation in PALS results in increased caregiver-responsive parenting behaviors relative to the control group and, if so, to what extent. The second secondary aim is to evaluate whether children of caregivers who receive the PALS intervention demonstrate improved self-regulation outcomes compared with children in the control group.

## 2. Methods

### 2.1. Study Design

This randomized controlled trial (RCT) evaluates whether increased parental responsiveness improves behavioral outcomes and normalizes atypical neural development. The study is conducted across multiple clinical and research sites, including the Children’s Learning Institute at UTHealth Houston and the University of Houston. Data collection occurred at the primary research sites and Baylor College of Medicine. This study is registered as a clinical trial on ClinicalTrials.gov (NCT04856501) before the enrollment of the first participant. All study procedures have been approved by the UTHealth Institutional Review Board, IRB number HSC-MS-17-0190, with an original approval date of 27 January 2021. Informed consent was obtained from all subjects involved in the study.

Participants in the study are randomized into one of two conditions: Play and Learning Strategies (PALS) intervention or active control.

PALS was developed in English and Spanish and offers materials that target parents of infants (PALS I) and parents of toddlers and young children (PALS II). The program is listed in four national What Works Clearinghouses and in the annual yearbook produced by the National Home Visiting Resource Center. This preventative positive parenting aims to support the relationship between parents and children while promoting language and cognitive skills and social development through 10 sessions lasting around 90 min each. The intervention utilizes techniques such as video modeling, feedback sessions, and assigned learning modules to facilitate caregiver participation in this highly effective parenting intervention. The accessibility of the PALS program is improved with adaptations like ePALS, which offer PALS online, making it easier for families dealing with obstacles to access PALS resources at home on their schedule. The ePALS program is structured across 10 sessions (Table 1), addressing key aspects of responsive parenting, and is conducted over 9 weeks, with each session lasting 90 min and sessions 6 and 7 combined in one week since they are both related to language.

Previous research guided the planning and the development of the research study. The research program was initiated in the summer of 2020 during the height of the COVID-19 pandemic. Despite the challenges posed by this period, the investigators chose not to delay enrollment, recognizing that 2020–2021 was a period of heightened isolation for families, making access to intervention and support services particularly critical. To maintain the safety of both enrolled families and PALS coaches, we implemented ePALS. Thus, the study seeks to assess the efficacy of ePALS, a digital adaptation of the PALS program, considering the pressing need to assist families of preterm toddlers and the capacity of responsive parenting to enhance neurodevelopmental outcomes. Utilizing digital technology, ePALS provides a scalable and accessible method to improve parental responsiveness, hence promoting enhanced behavioral and neurological outcomes for highly preterm toddlers [27]. Although initiating the study during the COVID-19 pandemic addressed an urgent need for remote support among families of very preterm children, pandemic-related stressors may have influenced recruitment, retention, and engagement with the intervention. Factors related to the pandemic likely contributed to variability in participation and session completion.

In this study, ePALS offers a parenting intervention featuring a 9-week, internet-based coaching program designed to enhance caregiver responsiveness and support child development. Parents receive weekly coaching sessions via video chat with a trained ePALS coach. These coaching sessions focus on contingent responsiveness, warm sensitivity, maintaining attention, and verbal scaffolding to promote cognitive and socio-emotional development in children. Parents practice strategies through interactive activities, video modeling, and self-reflection exercises. Each session requires parents to record and upload a short video of themselves engaging in structured play with their child, which is reviewed during coaching sessions. Participants in the ePALS group receive a tablet device to access intervention materials.

### 2.2. Active Control

Participants in the active control condition receive weekly phone calls and digital educational materials about typical child development milestones for 1–2-year-old children. The duration and frequency of interaction with a coach are designed to match the PALS group to ensure comparable engagement. Unlike the PALS group, control condition activities do not include coaching on responsive parenting techniques. Parents in this group do not receive a tablet device but have materials mailed to them to ensure accessibility for families without reliable internet access. The two conditions are designed to directly compare the impact of caregiver responsiveness coaching (PALS) versus general child development education (active control) on child executive function, emotion regulation, and neural development.

### 2.3. Trial Design, Randomization, and Recruitment

**Figure 1:**
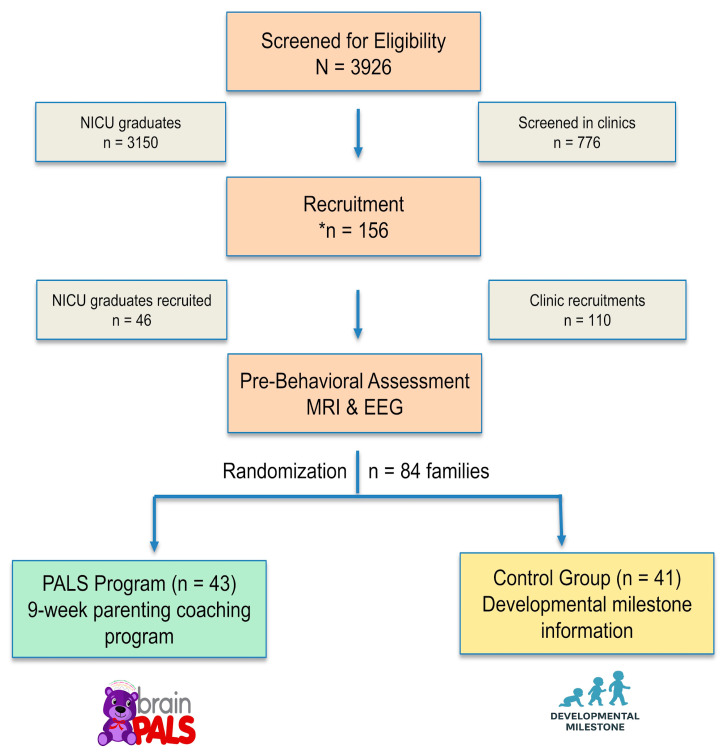
CONSORT (Consolidated Standards of Reporting Trials) flow diagram. *n does not include particiants recruited via Facebook advertisement or direct recruitment efforts.

This study is a parallel-group RCT utilizing stratified randomization to ensure balance across key variables, including sex, gestational age, and corrected age. Participants are randomly assigned to one of two groups: the intervention group receiving ePALS or the control group receiving developmental milestone information. The study enrolled parents and their toddlers born between 22 and 35 weeks of gestation and adjusted to 15–28 months of age at the time of enrollment. Recruitment is conducted through neonatal follow-up clinics and local outreach programs. Participant recruitment and enrollment are the key to any successful study, yet they remain one of the primary challenges for investigators. Although the research team for the current project is still enrolling participants, we have achieved the current enrollment of 156 participants through strong partnerships with UT Physicians Pediatric clinics and a neonatal intensive care unit (NICU) affiliated with Memorial Hermann Medical Group. Participants are recruited from the following sites: UTHealth Neonatal High-Risk Clinic—A comprehensive primary care clinic that provides routine follow-up care for very preterm (VPT) children from NICU discharge through age three, ensuring continuity of care for high-risk infants, and UT Physicians Primary Pediatric Care Clinic—A network of pediatric clinics with over 50,000 patient encounters annually, serving a diverse population, including children born preterm. This clinic provides general pediatric care while monitoring the developmental progress of preterm infants and toddlers. Based on current recruitment success rates, the research team approached 10 eligible families for every family enrolled in the study. During periods when there was a decrease in clinical recruitment, the research team expanded enrollment beyond our partnering clinics. Specifically, flyers were posted at community gathering locations and social media sites. Approximately 10% of the current sample was recruited using these alternative strategies. In the project’s fifth year, we started recruiting NICU graduates from Memorial Hermann Hospital as they transitioned to the EPIC system, which refers to the Epic Systems electronic health record (EHR) platform, which was used to identify potentially eligible participants and to extract relevant clinical and demographic information for recruitment and study monitoring, and successfully navigated multi-institutional IRB approvals. This collaboration allowed us to directly contact the eligible and high-priority population from the NICU clinic after the discharge by sending a letter or contacting them by phone or text, which has significantly increased our ability to screen a more eligible population. The flow diagram shows the total participants screened for eligibility, the recruited participants out of them, and the randomization shown in Figure 1. A total of 84 families were randomized to date; 43 were randomized to the PALS group, and 41 were randomized to the control group. Interestingly, we have three fathers who took part in the study and were randomized. Due to the small number of fathers participating, no conclusions can be drawn about differences in caregiver role engagement or outcomes. The sample demographics are presented in Table 2.

### 2.4. Eligibility Criteria and Participant Characteristics

Eligible participants for this study are toddlers considered VPT, or very premature (22–35 weeks gestation, corrected age 15–28 months), and their English and/or Spanish-speaking parents; however, primary analyses focus on children born very preterm (<33 weeks gestation), consistent with the study’s conceptual framework and primary aims. Ineligible participants include toddlers with severe congenital anomalies, toddlers unable to undergo an MRI or EEG scan (presence of metal-based medical implants, severe movement disorders, etc.), non-English or Spanish-speaking families, or families with limited access to the internet that would limit their ability to use study devices.

### 2.5. Sample Size Determination

A power analysis with 1000 replications was conducted to determine this study’s minimum detectable effect size (MDES). Based on expected intervention effects, a sample size of 160 (with 80 per group) was calculated to provide adequate power (80%) to detect an intervention main effect size of 0.36 between the PALs intervention and control across primary outcomes, including executive function, emotion regulation, and neural connectivity. The current analyses are based on a partial sample (n = 84) and are therefore underpowered for several planned behavioral and neurodevelopmental outcomes.

### 2.6. Study Outcomes

The study included 10 outcome measures, as outlined in Table 3. These measures were collected before and after the intervention.

Parent–child toy play [22] observation assesses the quality of parent–child interaction by conducting an 8 min play session. A standard set of toys consists of a shape box, a pig and pen pond playset, a bus with figures, and a puppet, and it is placed in an accessible area for parents and children. We then instruct the parent to play with the child as they typically would and to address any of the child’s needs throughout the session. The task is then recorded to capture both participants’ activity throughout the session.

In the waiting task [30], we place a brightly wrapped gift within the child’s reach, and at the same time, the parent is given questionnaires to fill out. We instruct the parent to explain to the child that the gift is a surprise for them, but they have to wait until the parent finishes the paperwork and are also instructed to avoid giving any food, toys, or any other object during that time and do the usual thing they do to make their child wait. We then set the timer for 8 min. The parents finish their questionnaires during this period and are asked to review their answers if they finish early. After 8 min, we signal the parent to let the child open the gift. The task continues even if the child cries but ends when the child shows significant distress.

Independent toy play [22] assesses the child’s ability and behavior to play alone with the toy. We provide the parent with a tablet to complete questionnaires in the meantime while we give the child four separate toys in sequence: 18 blocks and a bucket, a pop-up toy, a baby doll set (doll, bottle, spoon, chair), and a big red farm toy with figures. The toys are given to the child for about 3 min each. We provide the child with a brief demonstration of action (e.g., dumping blocks, activating pop-ups, simulating feeding the doll) before placing it within the child’s reach while saying, “See what you can do with these toys”. We repeat the action by giving the same prompt when the child stays away from the toy for ten seconds to help them focus on it again. The entire procedure is video recorded.

The 3-6-9 box task evaluates spatial working memory and search approaches through sets containing 3, 6, or 9 identical boxes with animal pictures on a lid. The assessment of levels (3-box, 6-box, 9-box) occurs sequentially based on the child’s performance. We place small rewards, such as Cheerios, into all boxes for the child to observe, saying “Watch me” during the first part of each level. A lid concealed the boxes before each search trial began. We then start a three-second countdown before revealing the boxes to tell the child, “Find one. Find a Cheerio”. During each trial, the child can open only one box at a time. The child could eat their discovered reward but had to try again when they select an empty box. We track each chosen box to note whether it contains a reward (correct) or an empty box that has already been searched and keep a record of this information. The task ends when the child selects the same empty box four times consecutively. The child needed to find all rewards within ten trials on the 3-box task and fourteen trials on the 6-box task to advance to the next level. The 6-box and 9-box levels had a maximum trial limit of twenty.

The gift wrap/waiting for bow task [22] consists of two parts. During Phase 1 (No Peeking, 1 min), we tell the child that we have a surprise for them, but we must wrap it first. We then go behind the child to make wrapping sounds by using tissue paper and empty bags in our hands while instructing the child to look ahead and “Try not to peek.” The child receives two warnings after looking at the present. During Phase 2 (Waiting for Bow, 2 min), we place a gift in front of the child and state that it is wrapped now, but we forgot the bow and need to get it. We tell the child to avoid touching or opening the present while we bring the bow. We then step out of sight for two minutes before returning to let the child open their gift.

The Consistent with previous work [28] evaluates problem-solving and tool use with minimal parental assistance, with each level allowing up to seven minutes for the child to attempt the task while being recorded from both front and side cameras. In level 1, we place a book inside a lockbox toy and demonstrate to the child the mechanism of wheel-based locking. After the demonstration, we ask the child to retrieve the book from the box. For the Level 2 task, we provide the child with a screwed stick and a shorter clear tube containing a small bath toy, which serves as a tool to retrieve the small bath toy placed inside the clear tube. The prompt given to the child is, “Can you get the toy out of the tube?” Level 3 used the same setup as Level 2, but this time we gave the child a larger tube with the small bath toy in between and an unscrewed stick. Before using it to retrieve the toy from the tube, we asked the child to screw the stick back together, which tested both tool construction ability and problem-solving capabilities. We tested the impacts of PALS with available preprocessed data collected during the Tool Task.

Magnetic Resonance Imaging (MRI)—this non-invasive imaging technique involves the collection of high-resolution anatomical images of the brain, which allows for examining structural development and evaluating neuroanatomical factors that offer insight into cognitive and behavioral outcomes in early childhood, and is done following the structural MRI and white matter development protocol. On the day of MRI acquisition, caregiver(s) and their child will arrive at the Baylor College of Medicine’s Core for Advanced Magnetic Resonance Imaging (CAMRI) facility, where a research assistant will direct the caregiver to a quiet room where they can soothe their toddler in preparation for sleep. The quiet room was made dark using blackout curtains, and an MRI-safe gurney was provided. Parents were instructed to have their child fall asleep and to notify the research staff when the child reaches deep sleep and is lying on their back. After the child was asleep, earplugs rated at NRR 33 and specifically designed for use with children (extremely soft and pliable) were inserted into the child’s ear. Then, researchers covered the child’s ears with MRI-safe hearing protective foam, which is heavily padded and wraps around the child’s head with a flexible elastic band. After the ear protection is secured, the child is moved into the bore of the MRI, where a member of our research team places one hand on the sleeping child, which we have previously found maintains sleep. If the child moves at all during image acquisition, the researcher stops the scan and waits for the child to return to a deep sleep. We have found this approach of pausing and restarting scan acquisition minimizes incomplete scanning sessions and the need for parents to return for multiple MRI visits. We acquired T1- and T2-weighted images (each approximately 4 min in duration) to quantify gray and white matter volumes, parcellate brain regions for regional volumetric analyses, and generate subject-specific ROIs for seed-to-seed structural and functional connectivity analyses. Diffusion tensor imaging (DTI; 5 min) was collected to assess structural connectivity. Although not part of the original study design, when toddlers remained asleep following DTI acquisition, we also obtained resting-state functional MRI to evaluate functional connectivity. The collected data were preprocessed using the standard FSL (FMRIB Software Library, https://fsl.fmrib.ox.ac.uk/fsl/docs/, accessed on 1 April 2024) pipeline. We used the Infant Brain Extraction and Analysis Toolbox (IBEAT) to parcellate 90 anatomical brain regions using the UNC atlas that was closest to the child’s age (i.e., 12 months, 18 months, 24 months).

EEG—This developmentally sensitive imaging tool captures electrical activity in the brain in real-time, enabling observation of neural responses to behavior. Thus, this technique can provide insight into the neurodevelopmental trajectories of social, emotional, and cognitive processes. EEG captures both ongoing brain oscillations that are time-locked to behavioral conditions and event-related potentials (ERPs), which are transient deflections in brain activity time-locked to specific stimuli [31].

To collect this data, an EEG was recorded using a 64-electrode actiCap system (Brain Products GmbH). During data collection, the sampling rate was set to 1000 Hz, and the reference electrode was initially located at FCz; however, it was re-referenced at the processing steps. EEG recordings were conducted in Brain Vision Recording software (Brain Products, Munich, Germany, version 1.25.0201).

EEG setup and recording involve children sitting on their caregiver’s lap. First, EEG cap sizes are selected based on the child’s head circumference. After the cap is selected, EEG electrode bundles are populated on the cap, and saline conductive gel is secreted under each electrode using a syringe. The gel is water-soluble, allowing it to be easily cleaned with a washcloth after the session. Once the gel is applied, the cap is placed on the child’s head, and then the caregiver and child are brought to the testing area. The testing area is a sound-attenuated chamber, where the caregiver is directed to sit in a chair in front of a computer monitor, and the child sits on their caregiver’s lap. The computer monitor was approximately 21 inches by 12 inches, and the chair was placed about 50 inches in front of the monitor. While the child acclimates to the cap and the testing environment, the research assistant examines the impedances (scalp-electrode resistance). Electrodes with impedances above the threshold (10–20 kΩ) were manually adjusted to improve connection with the scalp and/or reapplied with gel. Once impedance thresholds had been met for all electrodes, recording could begin. Before the first task begins, parents are instructed to continue holding their child on their lap for the duration of the data collection. However, they are advised not to interact directly with their child during testing. Once the first task is ready to begin, one research assistant remains in the testing chamber with the caregiver and child. In contrast, a second research assistant remains outside of the testing chamber and controls the acquisition software and camera that records the child’s face and monitor. The task stimuli and the child’s face are recorded using a single camera positioned directly in front of the child. A mirror placed behind the child reflects the stimuli presented on the monitor in front of them, allowing both the child and the stimuli to be captured simultaneously in the same camera view.

EEG tasks—there are three total recording periods during each session.

Resting-State Recording: Each session included a 3 min resting-state recording during which the child’s neural activity was measured at rest. To maintain a calm yet visually controlled environment, a research assistant inside the testing chamber quietly rotated a bingo ball spinner containing small toys. Parents were instructed to remain silent but to quietly prevent the child from grabbing the bingo ball spinner if they tried to do so. The research assistant remains silent throughout the recording.

Visual Attention Paradigm: A visual attention task adapted from Richards (1997) [32] was presented using Presentation software (Neurobehavioral Systems, Inc., Berkeley, CA, USA, https://www.neurobs.com/). Children passively viewed movie clips of Sesame Street characters. Each clip appeared in 4 × 2-inch windows on either the left or right side of the screen and either remained stationary or moved across the screen. Each trial lasted 8–12 s, during which the video clips disappeared, reappeared, or shifted direction. Each trial included instrumental background music that stopped and restarted at trial onset. The paradigm consisted of five blocks of 14 trials each, with full-screen attention grabbers between each block. The entire task lasted approximately 12 min. Parents were instructed to remain silent to minimize distraction, and research assistants quietly redirected children’s attention to the screen when necessary.

Dynamic Face Task: For the face-processing paradigm, EEG was recorded while toddlers passively viewed 150 dynamic facial expressions that changed from neutral to angry, neutral to happy, or remained neutral throughout. Each trial consisted of a 1000 ms video depicting one of the three expression types. The videos were specifically created for this task, with each expression reaching peak valence at 500 ms and remaining static for the final 500 ms of the trial. Neutral expressions remained static for the entire duration. To create the stimuli, four female volunteers of different racial and ethnic backgrounds (Black, White, Hispanic, and Asian) were recruited from the Department of Psychology at the University of Houston to record videos that contain each of the facial expression conditions. These videos were then produced following protocols established in prior developmental facial emotion studies. Face-sensitive ERPs are shown to be modulated by myriad features, some of which concern bottom-up properties related to visual contrast, and others that may reflect familiarity based on gender, age and race/ethnicity [32]. To mitigate effects of familiarity due to “other gender, age, and race effects” children viewed stimuli of female faces that matched their race and ethnic and cultural background [33].

Electroencephalogram (EEG) preprocessing: Preprocessing is conducted using custom MATLAB (Matlab Version R2021b) scripts adapted from the Batch Electroencephalography Automated Processing Platform (BEAPP) and the Harvard Automated Processing Pipeline for EEG (HAPPE) modules [34,35]. For each EEG task recording, epochs that exceeded ±200 μV or contained residual artifacts were excluded, and poor-quality channels were interpolated using spherical spline interpolation. Participants with at least 20 artifact-free epochs per condition were included in analyses. Prior to automated processing, raw EEG files are visually inspected to identify and exclude channels or segments with systematic artifacts. Following inspection, data are imported into MATLAB and processed through the BEAPP pipeline. The preprocessing pipeline includes the following steps: (1) down-sampling to 250 Hz; (2) removal of 60 Hz line noise using the CleanLine algorithm within the Prep module; (3) application of a 1 Hz high-pass and 100 Hz low-pass filter; and (4) standardization of sampling rates across participants. Independent Component Analysis (ICA) is then performed using the HAPPE module, and artifacts are automatically classified and rejected using the MARA algorithm. The cleaned data are re-referenced to the average across all scalp electrodes. Finally, linear detrending is applied to remove slow drifts, and event-related epochs are segmented based on task events and research questions.

### 2.7. Secondary Outcome Measurements and Covariates

Additional outcomes include parental mental health measured by Beck’s Depression Inventory (BDI), home environment measured by Confusion, Hubbub, and Order Scale (CHAOS), child’s early language and communication development using MacArthur-Bates, and outpatients’ therapies.

BDI—a tool used to assess depressive symptoms from minimal to severe levels; parents experiencing depression were enrolled in this study because PALS has demonstrated efficacy in improving parenting outcomes even among caregivers experiencing major depression [36,37].

CHAOS—this scale measures environmental confusion in the home, such as noise, crowding, and disorganization. This is associated with both parenting behavior and child developmental outcomes [38,39].

MacArthur-Bates—this parent report instrument collects the data to determine whether improved parenting responsiveness leads to early childhood language and communication development [40].

Outpatient therapies—this instrument collects the data about a child receiving any therapy, including physical, occupational, speech, feeding or any other therapy (please see Supplementary Materials).

COVID-19 Exposure and Family Impact Survey (CEFIS)—this instrument collects data about the impact of COVID-19 on the family, specifically the main caregivers [41].

### 2.8. Adherence to the Study Protocol and Intervention

Adherence to the study protocol and intervention procedures is established through multiple aspects of the research design. The PALS intervention sessions are logged and monitored by coaches to ensure full completion of all nine weekly sessions. Video uploads from parents in the PALS group are reviewed in weekly coaching feedback sessions that include all PALS coaches, the interventionist (CP) and the founder of the PALS program (SL). The active control group participants confirm their weekly engagement with educational materials and coaching calls.

### 2.9. Data Sources, Collection, and Validity

To ensure high-quality measurements, data is collected from multiple sources, including behavioral assessments, MRI and EEG data, and patient-reported data. Behavioral assessments are conducted at all three stages of the study: pre-test (baseline), post-test 1 (UTH behavioral), and post-test 2 (UTH/UH). Time between post-test 1 and post-test 2 in months (M = 2.78, SD = 2.03). Sessions are video-recorded and coded using established systems. MRI is conducted during a toddler’s natural sleep at Baylor College of Medicine to ensure valid structural and functional connectivity measures. EEG imaging is performed at Dr. Bick’s lab at the University of Houston to measure event-related activity and sustain attention. To assess the integrity of white matter structure, DTI data is obtained. This data is processed using a standard analysis pipeline in FSL to minimize bias. Additional data sources include parent self-reports and medical record reviews. Parent-reported data is received through surveys and questionnaires collected at all 3 timepoints. Medical history, including birth weight, gestational age at birth, gender, postnatal steroid administration, and bronchopulmonary dysplasia and maternal depression, is established from medical record review. This data is used to classify the sample and as possible covariates in the case of unhappy randomization.

### 2.10. PALS Exit Interview

Following completion of the PALS intervention, caregivers completed brief telephone exit interviews. Interviews were administered by trained study staff and included open-ended questions assessing perceived changes in parenting behaviors, child development, helpfulness of specific PALSs, overall satisfaction with the program, and recommendations for program improvement.

Responses were reviewed to identify recurring themes, which are presented in Section 3 to complement quantitative findings and provide contextual insight into caregiver experiences with the intervention.

### 2.11. Planned Analyses

The following analyses are planned for the trial and will be conducted after data collection is complete. Descriptive statistics will be generated for all parent and child measures. Planned parental responsiveness variables include contingent responsiveness, warm sensitivity, verbal scaffolding, and maintaining versus redirecting attention. Planned child self-regulation measures include executive functions (working memory, inhibition, problem solving, and attention) and emotion regulation. Medical and psychosocial factors previously associated with neurodevelopmental outcomes in very preterm [42] will be evaluated as potential covariates. Medical variables will include birth weight, gestational age, gender, postnatal steroid exposure, bronchopulmonary dysplasia, and prior or current outpatient therapies. Psychosocial variables will include race and ethnicity, primary home language, maternal education, social support, maternal depressive symptoms, home environment quality, and parental developmental beliefs. All data will be inspected for accuracy and outliers by the analytic team once collection is finalized. Extreme values that cannot be reconciled will be flagged, and primary analyses will be conducted both with and without these data points to assess their influence. Residual distributions will be evaluated to determine whether non-linear models are warranted; Poisson or negative binomial models will be considered as appropriate, likely implemented via PROC GENMOD in SAS (version 9.4). Planned analyses will also examine hemispheric differences in neural regions of interest using t-tests; if significant, hemisphere will be included as a factor in primary models. Prior to hypothesis testing, we will determine whether data reduction is indicated. Consistent with previous work [43], indicators of early executive function and emotion regulation may load onto one- or two-factor models. Intervention effects on parental responsiveness will be tested using 2 (group: ePALS vs. control) × 2 (time: pre vs. post) repeated measures ANCOVAs. The planned primary effect of interest is the Group × Time interaction, which evaluates whether change over time differs between groups while adjusting for baseline differences. Parallel 2 × 2 repeated-measures ANCOVAs are planned for child behavioral outcomes and child brain development. Following primary intervention tests, we will explore whether covariates moderate intervention effects, and significant pathways identified in earlier aims will be evaluated as potential mediators. Planned mediation analyses will be conducted separately for behavioral and neural outcomes within an SEM framework using Monte Carlo bootstrapped estimation of direct and indirect effects [44].

## 3. Results

### 3.1. Moderating Influences

Some potential factors may moderate intervention effects. In regard to the toddler subject, gestational age (whether EP or VP) may moderate the impact, as well as baseline executive function and emotional regulation scores. In regard to the parents, moderators include scores on the BDI, CHAOS, MacArthur-Bates, outpatient therapies and CEFIS.

### 3.2. Data Management

Study data is stored in secure files accessible by authorized research staff. Data are de-identified. Trained research assistants who are blind to the group allocation code behavioral data while neuroimaging data is processed using standardized pipelines such as iBeat and FSL. To protect participant confidentiality, all identifiable information is replaced with unique study IDs. In compliance with NIH open-access policies, final datasets are deposited in publicly accessible repositories, such as the NIH Pediatric MRI Data Repository. Study findings are disseminated through peer-reviewed publications and conference presentations, ensuring transparency and contributing to the broader scientific community.

### 3.3. Statistical Analysis

To evaluate the impact of attrition, sensitivity analyses compared baseline characteristics of participants who completed follow-up assessments with those who did not. Analyses also accounted for the fact that repeated measurements were collected from the same participants and that some families contributed more than one observation, using appropriate within-subject correlation structures. Primary analyses focused on changes over time and differences between groups in these changes. Because recruitment and data collection are ongoing and the current sample represents an interim cohort, all analyses were considered exploratory and intended to generate hypotheses rather than definitive conclusions.

Planned ANCOVA models will compare post-test outcomes across conditions, adjusting for baseline measures. A Bonferroni correction is applied to account for multiple comparisons and control for Type I error. Additionally, mediation and moderation models are used to examine the role of maternal responsiveness in predicting child neurodevelopmental outcomes.

The anticipated attrition rate of around 10% was exceeded, fluctuating between 20% and 40%. The majority of participants’ losses occurred during baseline evaluation. Retention efforts focus on frequent follow-ups, flexible scheduling, and engagement strategies to maximize retention and maintain statistical power for primary analyses. Even with flexible timing, online tests, follow-up calls, and regular updates for parents, people still left the study. Despite these efforts, attrition may introduce bias; therefore, findings should be interpreted with caution. A sensitivity analysis will be conducted to address missing data.

### 3.4. Preliminary Findings

#### 3.4.1. ePALS Session Retention Rate

**Figure 2:**
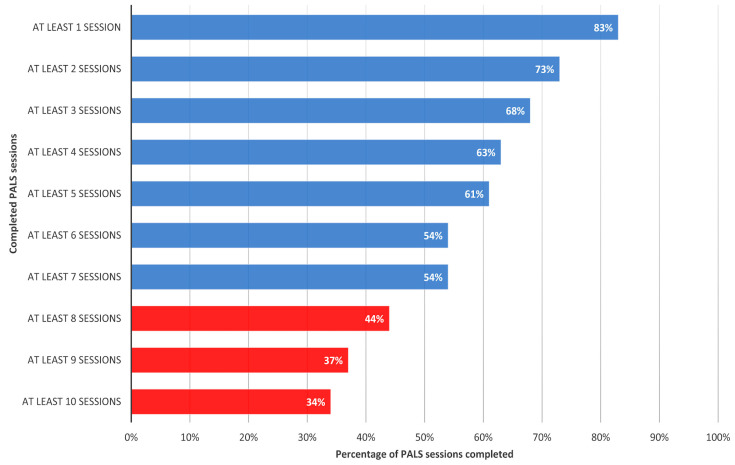
ePALS session retention rate.

For families randomized to PALS, 83% of families completed at least one session of PALS, 73% of the families completed at least two sessions, 68% of the families completed at least three sessions, 63% of the families completed at least four sessions, 61% of the families completed at least five sessions, 54% of the families completed six sessions and also 54% completed seven sessions. Additionally, 44% of the families completed at least 8 sessions, 37% completed at least 9 sessions, and 34% completed all 10 sessions (see Figure 2). Completing seven sessions is considered a completed PALS intervention for families who struggle to complete them. However, those who complete the session will be offered the next session and encouraged by their coach to complete the remaining sessions. For example, if a family completes seven sessions, they are offered the eighth; similarly, if they complete the eighth, they are offered the nineth. All the families are encouraged to complete all 10 sessions.

#### 3.4.2. Tool Task Findings

**Figure 3:**
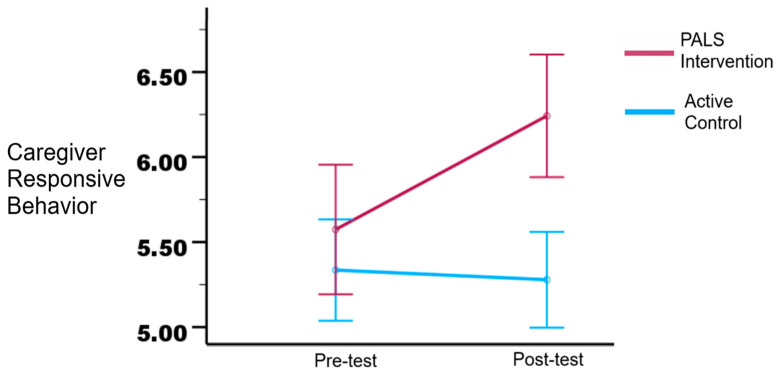
Pre-test and post-test observed parent behavior during the tool task. The red line represents families assigned to the PALS intervention, and the blue line represents families assigned to the control group.

Our preliminary findings demonstrate a clear positive impact of participation in the PALS parenting intervention on parental responsiveness (see Figure 3). Specifically, a repeated-measures ANOVA examining observed parent behavior during the tool task, including measures of supportive presence and quality of assistance, indicated a significant effect of the PALS intervention, F(2, 30) = 6.07, *p* = 0.036. Parents who received the PALS intervention demonstrated greater improvements in parental responsiveness from pre-test to post-test compared to control families. This analysis includes all families with more than three completed PALS or active control sessions. For repeated-measures analyses, participants who completed more than three intervention sessions were included. This criterion ensured minimal exposure to the intervention sufficient to plausibly influence early outcome measures and reflected the distribution of session attendance within the current sample and also showed an early effect. Families were encouraged to complete all ten sessions whenever possible.

At the end of the PALS program, the PALS coach conducted a brief exit interview with parents. Table 4 includes quotes from parents provided during this exit interview that represent general themes that emerged during these interviews.

## 4. Discussion

In summary, this research protocol outlines a randomized controlled trial of ePALS, a web-based parenting intervention that delivers remote coaching to increase responsive caregiving in families of preterm toddlers. The study addresses a critical gap in early intervention by using technology to overcome access barriers and support families facing elevated developmental risks. It evaluates the potential impact of ePALS on parental responsiveness and child self-regulation through both behavioral and neurodevelopmental assessments, while also examining implementation and retention. By integrating these components, the trial offers a comprehensive view of the possible effectiveness and feasibility of ePALS, informing the scalability of remotely delivered parenting interventions for high-risk populations.

Findings from the current ePALS trial, conducted during and after the COVID-19 pandemic, highlight important changes in caregiver engagement. It is well documented in the literature that the COVID-19 pandemic increased family stress and disrupted family daily functioning through altered routines and loss of income streams and structural supports, such as childcare [45]. Although the impact of COVID-19 unfolded across a period of 1–2 years, there may have been a difference in relative desire to enroll in research overall and parenting programs specifically, which may have impacted recruitment and retention in this research. Specifically, in our current research cohort, there was a relative decline in the number of families completing all or even the majority of PALS sessions. Among the 43 families assigned to the intervention condition, approximately half completed at least seven ePALS sessions, which was the minimum threshold considered sufficient to achieve core benefits of the program. However, only one-third of participants completed all ten sessions. This level of engagement is notably lower compared to PALS session completion rates observed in prior implementations of the PALS program. For instance, in a previous study [22], 68.4% of families completed the sessions, with an overall attrition rate of 32%. These differences in completion rates highlight essential considerations for adapting and delivering parenting interventions in virtual formats. It is important to note that the current trial and earlier implementations [10] are not directly comparable, as ePALS was delivered remotely through a web-based platform, but they are very similar. In contrast, the earlier PALS programs were conducted in-person with face-to-face coaching. Whereas comparisons between in-person and virtual formats should be made cautiously due to fundamental differences in delivery mode, it was anticipated that the increased accessibility of ePALS, through online materials and remote coaching, would facilitate higher completion rates.

On one hand, we did not observe retention comparable to prior implementations of PALS, either in-person or virtual. Although a similar study [27] successfully implemented an electronic version of PALS with parents of 3–9-month-old infants, interventions reported increased flexibility in how and when coaching sessions were delivered. However, this advantage may not have translated to the current study. One possibility is that the remote format in this context did not foster the same level of social engagement and trust achieved in prior virtual implementations. An alternative, and not mutually exclusive, explanation is that the perceived burden of participation was higher, with digital access demands, caregiver stress, and competing responsibilities negatively impacting retention. Because concurrent data directly comparing virtual to in-person or experimental manipulation of PASL dosage is not available, these interpretations on implementation factors remain highly tentative. Importantly, there remains a need for further research to optimize engagement and adherence in telehealth-delivered parenting interventions, particularly for high-risk populations. There also remains a gap in our understanding of how to develop more streamlined intervention models that reduce session burden while maintaining effectiveness to better align with caregivers’ post-pandemic realities.

Given the pattern of participation, evaluating outcomes after three sessions offers an opportunity to assess the early impact of the intervention. Since a substantial portion of families, 68%, complete at least three sessions, this point represents a practical and informative exposure threshold. Assessing early effects also allows us to explore the minimum effective dose of the intervention, which is especially important in populations where completing all sessions may not be feasible due to structural or contextual barriers. Moreover, since families are continuously offered subsequent sessions regardless of how many they have completed, this flexible model supports examining intervention effects at multiple stages. By analyzing outcomes at the three-session mark, we can better understand early responsiveness and inform intervention delivery strategies, with considerations for generalizability.

Importantly, although the current implementation of ePALS did not achieve the expected dosage, there are nonetheless statistically significant associations with behavior and outcome. Preliminary results of the Tool Task observational assessment suggest that families who received the intervention show significantly higher parental responsiveness. It is important to consider these findings with caution because the included data reflect a subset of the final study N. This is the first instance that the tool task has been used to evaluate PALS intervention effects, such that in previous implementations, PALS effects were evident during parent–child observation of a free-play session. By identifying the PALS effects during the tool task, this research suggests that PALS may influence parent–child interaction during problem solving, where parents were explicitly asked to support their child in accomplishing a goal that was just beyond the child’s current ability. The initial findings suggest that ePALS shows both feasibility and effectiveness in association with more positive parenting behaviors among vulnerable families. Future follow-up assessments will evaluate the long-term maintenance of these results and their connection to child neurodevelopment.

Parent perspectives on PALS highlighted the program’s strengths and opportunities for improvement. After completing the PALS program, coaches conducted brief exit interviews with participating parents to gather qualitative feedback on their experiences. Table 4 presents representative quotes that illustrate recurring themes across these interviews. Overall, parents reported that the program helped them develop core responsive parenting skills. These included following the child’s lead, using positive affect, and promoting language-rich interactions. Many described the sessions as empowering, particularly in reinforcing their role as active participants in their child’s development. At the same time, parental feedback highlighted further opportunities to improve the PALS program’s reach and impact. Several parents noted that families facing greater adversity, such as those with a history of trauma, limited financial resources, or housing instability, might require additional support to benefit from the program fully. Suggestions included expanding the training of PALS coaches to include trauma-informed practices and equipping them with knowledge of how to help families access local nutrition, mental health, or housing services. This aligns with broader calls to embed parenting interventions within a more holistic support system that addresses social determinants of health. Additionally, parents strongly desire more flexible and sustained access to PALS materials. While they valued the coaching sessions, many indicated that having open access to digital resources, including video demonstrations and coaching guides, would reinforce the strategies introduced and allow them to revisit content as needed. Expanding public access to PALS materials could also benefit other caregivers in the home, such as grandparents or partners, who may not have participated in coaching sessions. Although parents did not explicitly suggest this, broader accessibility has the potential to support more consistent caregiving practices across the family unit. These findings emphasize the importance of implementing science frameworks and community-based research strategies to inform the next program development phase. Making the program more accessible, flexible, and contextually responsive can help ensure that PALS continues to meet the needs of diverse families, particularly those facing structural barriers to engagement. Future iterations of PALS could benefit from user-centered design approaches and partnerships with community organizations to tailor support for families navigating complex life circumstances.

Recognizing that PALS is one of several evidence-based programs that strengthen responsive parenting and promote positive parent–child interactions is essential. For example, Attachment and Biobehavioral Catch-up (ABC) [24,43,46] is a well-established intervention that provides in-home, real-time coaching to help caregivers become more sensitive and responsive to their child’s cues. ABC is particularly effective for families facing adversity and has been shown to improve parenting behaviors and child developmental outcomes across multiple domains. Programs like ABC and PALS are part of a broader movement toward accessible, family-centered interventions that support early relational health as a foundation for lifelong development. Research also highlights the value of integrating mental health support into parenting programs. Combining responsive caregiving strategies with approaches such as cognitive behavioral therapy may help caregivers manage stress, reduce depressive symptoms, and foster more positive interactions with their children. This dual-focus model not only supports maternal mental health but may also lead to more robust developmental gains for children, underscoring the potential of integrated approaches to maximize impact for families facing elevated risk.

This research has several important limitations. The current results are based on limited outcome data because data collection and outcome coding are still ongoing. In addition, attrition remains an important concern. We were unable to speak with families who stopped participating or withdrew from the program because it became challenging to contact parents after they left the program. A final limitation related to attrition is that the ePALS coach conducted exit interviews, which could have led parents to provide more positive responses. We plan to implement simpler assessment methods, such as brief phone or text-based surveys, to determine the reasons families stop participating in the future.

Electronic resources are an additional limitation of the study, such that the intervention group received tablets connected to the internet, but the active control group did not receive any devices. The distribution of tablets between the intervention and active control groups might have affected how parents perceived the program and their level of participation. The digital exclusion issue persisted as a challenge because families with restricted digital literacy or unstable internet access might have found it difficult to fully participate in ePALS.

As noted above, we evaluated intervention-related effects for all participants who completed three or more PALS sessions. On one hand, including families who completed less than half of the intervention could be seen as a limitation, as the full effects of the program might be eclipsed by participants who completed very few PALS sessions. On the other hand, this approach is also conservative because detecting intervention effects even at lower doses and among families who faced barriers to completing PALS suggests that the program is likely effective for a broad range of caregivers. The full analysis will present all outcomes while focusing on families who finish at least seven PALS sessions, which represents a completed intervention.

It is also important to acknowledge that the study included three fathers, all of whom received the PALS intervention. Since the majority of caregivers who enrolled in this study indicated their role as mother, it was not feasible to include caregiver role as a feature of randomization, which could have resulted in a more equal distribution of fathers across control and intervention conditions. Moreover, the limited number of fathers enrolled in the study restricts the ability to generalize these findings to fathers.

The protocol described here and the preliminary research findings underscore the critical role of structured parental guidance in supporting optimal child development, particularly for children born at high medical risk. Promising intervention components and outcomes observed in the ePALS trial add to the growing body of evidence demonstrating the effectiveness of targeted, relationship-based parenting interventions. Importantly, the results suggest that similar gains could be achieved through responsive parenting programs adapted for families with limited access to traditional services, those with a low caregiver burden, and increased access to supportive services. As one of the first studies to deliver PALS through a fully virtual model, this trial provides valuable insights into the promise and challenges of scaling parenting interventions using digital platforms. By focusing on behavioral and neurodevelopmental outcomes, ePALS findings will advance our understanding of how early caregiving shapes developmental trajectories and inform the adaptation of evidence-based programs to meet the evolving needs of diverse and underserved families. Future research should continue exploring how remote delivery, implementation support, and family-centered adaptations can maximize engagement and impact, paving the way for broader public health applications and more equitable access to early developmental support.

## Figures and Tables

**Table 1:** List of concepts for each of the 10 ePALS sessions.

Sessions	Description
Session 1: Introduction.	The coach builds rapport and gathers information on family routines, parent expectations/beliefs, and community connections.
Session 2: Reading Children’s Signals.	The coach builds rapport with the parents. Parents begin to develop new skills in recognizing and understanding how to interpret the meaning of a child’s positive and negative verbal and nonverbal signals as a means of communication.
Session 3: Warm Responsiveness.	Responding to Signals—Parents learn to respond promptly and sensitively in ways that are directly linked to the child’s signals.
Session 4: Labeling Objects and Actions.	Parents learn how to incorporate rich language into interactions with their children with an emphasis on the use of labels for objects and actions.
Session 5: Maintaining Children’s Focus Of Attention.	Parents work to understand how to maintain children’s attentional focus and build interest rather than redirecting attention.
Session 6: Reading to Young Children.	Parents learn reading strategies that promote positive shared book-reading interactions with toddlers.
Session 7: Linking Objects and Actions.	Parents further develop strategies for using rich language with an emphasis on verbal scaffolding during play and daily routines.
Session 8: Guiding Children’s Behavior	Parents learn behavioral strategies (use explanations, turn tasks into games, give reminders, give choices, and give step-by-step directions) to help children focus their attention and manage their emotions and behavior.
Session 9: Daily routines.	Parents establish an understanding of how to integrate responsive behaviors together in everyday situations (baths, meals, dressing).
Session 10: Advanced Language Strategies.	Parents learn strategies to enhance their child’s language development further.

**Table 2:** Descriptive characteristics of randomized participants in PALS (n = 43) and control group (n = 41).

Sample Characteristics	PALS Intervention n (%)	Control Group n (%)
Child Sex		
Male	22 (51.2)	22(53.7)
Female	21 (48.8)	19(46.3)
Child Race		
Black or African American	14 (32.6)	13 (31.7)
White	21 (48.8)	18 (43.9)
Asian	2 (4.6)	2 (4.9)
American Indian or Alaska Native	-	2 (4.9)
Native Hawaiian or Other Pacific Islander	-	1 (2.4)
Declined to respond	6 (14)	5 (12.2)
Child Ethnicity		
Yes, Hispanic or Latino	24 (55.8)	23 (56.1)
No, not Hispanic or Latino	18 (41.9)	18 (43.9)
Declined to respond	1 (2.3)	-
Gestation Age Classification		
Extreme Preterm (22–27)	16 (37.2)	14 (34.1)
Very Preterm (28–33)	12 (27.9)	10 (24.4)
Late Preterm (34–36)	15 (34.9)	17 (41.5)
Caregiver’s relationship to Child		
Mother	40 (93)	41 (100)
Father	3 (7)	-
Other	-	-
Caregiver Education		
Primary School, Finished 5th Grade	1 (2.3)	1 (2.4)
Middle School	-	1 (2.4)
Some High School	3 (7)	1 (2.4)
High School Diploma or GED	6 (14)	8 (19.5)
Vocational or technical training	3 (7)	1 (2.4)
Some college but no degree	7 (16.3)	7 (17.1)
Associate’s degree (AA)	5 (11.6)	2 (4.9)
Bachelor’s degree (BA/BS)	8 (18.6)	11 (26.8)
Master’s degree (MA, MS, JD)	4 (9.3)	4 (9.8)
Other	-	1 (2.4)
Declined to respond	3 (7)	2 (4.9)
Caregivers’ household income		
200,001 or more	1 (2.3)	4 (9.8)
175,001 to 200,000	-	1 (2.4)
150,001 to 175,000	2 (4.7)	-
125,001 to 150,000	1 (2.3)	4 (9.8)
100,001 to 125,000	1 (2.3)	4 (9.8)
75,001 to 100,000	4 (9.3)	2 (4.9)
50,001 to 75,000	13 (30.2)	5 (12.2)
Less than 50,000	14 (32.6)	7 (17.1)
Declined to respond	7 (16.3)	14 (34.1)
	PALS intervention Mean (SD)	Control group Mean (SD)
Child Gestational Age (months)	20.59 (5.13)	19.67 (4.92)
Gestational Age at Birth(weeks)	30.2 (4.79)	30.75 (4.8)
Caregiver Age (years)	33.77(5.75)	32.61(7.49)

**Table 3:** Description of collected outcome measures.

Measure	Description	Parent Outcomes	Child Outcomes
Tool Task [28]	Child solves the problems using the tools given in increasing order of difficulty	Supportive presence and quality of assistance, caregiver emotional support, and problem-solving guidance during the task	Child Self-regulation
Waiting for Bow [22]	Child is asked not to peek while the gift is wrapped and waits before opening the gift	None	Child Self-regulation
Independent toy play [22]	Child plays independently with four different toys for 3 min each	None	Child Self-regulation
Parent–child toy play [22]	Parent and child play together with a set of 4 toys for 8 min	Display of positive affect, warmth, responsiveness, physical intrusiveness, negativity, verbal teaching, quality of verbal content	Child Self-regulation
3-6-9 box [29]	Child searches for a grid of boxes baited with lures. Grid increases from 3 to 9 boxes	None	Child Self-regulation
Waiting task [30]	Child is told to wait for 8 min as the parents are filling out the questionnaire	Parental use of verbal distraction, physical soothing, promotion, prevention, and harsh/ destructive response	Child Self-regulation
Regional grey matter volume (T1 & T2)	MRI scan assesses the brain structures and volume	None	Assessment of white matter integrity, neural correlations of executive function, and emotional regulation
Structural Connectivity (DTI)	Diffusion images evaluate the white matter pathways in the brain	None	Assessment of white matter structural integrity and connectivity
Resting state [31]	Measuring the brain activity in the resting state using EEG	None	Baseline neural activity and functional connectivity
Emotion processing task (ERP) [31]	Recording the brain’s response to the emotional stimulus with an EEG	None	Assessment of neural reactivity to emotional stimuli
Visual Attention Paradigm (EEG)	Recording neural oscillations during visually guided attention using attention eliciting video clips	None	Assessment of oscillatory modulation across distinct phases of visual attention

Executive and emotional regulation measures include the parent–child toy play observation, the waiting task, independent toy play, the 3-6-9 box task, the gift wrap/waiting for bow task, and the tool task. Measures of neural connectivity will include magnetic resonance imaging (MRI) and electroencephalogram imaging.

**Table 4:** Parents’ quotes from PALS exit interviews.

Interview Question	Parent Responses
Q. How has Pals changed your Parenting Behavior?	Parent 1—“It definitely helped me refine how I speak to the child and like, what sort of explanations that I need to help him explain concepts. But definitely helping me with tailoring my language towards him and putting a lot of more emphasis on saying positive things in a positive way.” Parent 2—“I feel like it’s made me a more attentive parent. So, just paying attention to my baby’s signals, the positive, negative signals, and then also even the unclear ones. Just give her time to really tell me what she’s trying to. To communicate whatever, she’s trying to say to me. So, it’s really made me attentive in that way. I feel like I have better skills to like, learn with her, to play with her, to interact with her on a daily basis, and that has made me a better parent.
Q. How has your child changed from the beginning of the program until now?	Parent 1—“He is definitely starting to say a lot more words. You ask him to say something he’ll make an attempt now before he even wouldn’t. And he’s gotten a little bit better about managing his emotions, and not just immediately throwing things. But yeah, it’s still a struggle.” Parent 2—“I think she definitely likes when I respond to her signals. I feel like she’s better able to communicate with me as well. Also, she signals a lot more. She’s more free to explore as much as possible. She feels confident exploring, like, whatever she wants to play with, without trying to get attention to. So, just making her more confident and free to play and explore. And, like, you know? figure out what her interests are, basically, so I enjoy seeing her grow in that way. Yeah.”
Q. And then, would you recommend the Pals program to other parents and why?	Parent 1—“Yes, I think it’s a really good way to help you learn to interact with your child in a way that you can get what you need across, and then they can understand it and help you with the teaching strategies.” Parent 2—“Yeah, yeah, I would because I feel like it gives parents a great toolbox of tools and skills to become good parents, not just like the early childhood phase of their baby’s life, but like long term because it helps you pay attention. Communicate well with your child. Interact with them. I’ll tell you, it just makes you a better parent overall, and makes you more sensitive to them and their needs, like it helps you validate their needs and understand that. They’re like individuals that have feelings. They have their own thoughts and feelings and desires. They have feelings now, and it makes you more attentive to those feelings and their needs, so I would definitely recommend it to other people. Yeah, well, thank you so much for being kind of friends.”
Q. Regarding the PALS sessions, which of the PALS strategies have been most helpful for you?	Parent 3—“All of them were marvelous. If my child is not focused, the strategies keep him interested. I didn’t know I needed to spend time with each child.” “PALS helped me to become a more knowledgeable mom.”
Q: What could we change to make this program better for families in the future?	Parent 4—“Have the ability to access the program on my personal equipment.” “Provide more resources for families that are in low-income situations.” Parent 5—“I want more sessions after the program is done. I want to continue learning and meet other parents, too.”

## Data Availability

The data presented in this study are not public and will not be available due to ethical restrictions and participant confidentiality. De-identified data will be made available upon completion of research activities in compliance with NIH current data sharing requirements, subject to institutional and IRB approval.

## Supplementary Materials

The following supporting information can be downloaded at: https://www.mdpi.com/article/10.3390/children13060774/s1.

## Abbreviations

The following abbreviations are used in this manuscript:

PALS	Play and Learning Strategies
ePALS	Digital version of Play and Learning Strategies
VPT	Very preterm
EF	Executive function
ER	Emotion regulation
NICU	Neonatal intensive care unit
RCT	Randomized controlled trial
VLBW	Very low birth weight
MDES	Minimum detectable effect size
CHAOS	Confusion, Hubbub, and Order Scale
ABC	Attachment and Biobehavioral Catch-up
