# Supplementary material for: Protocol and Preliminary Findings from the BrainPALS Study in Very Preterm Children: A Randomized Controlled Trial of a Digital Parenting Program
Source: Children (Basel). 2026 Jun 2;13(6):774. doi: 10.3390/children13060774 (PMC13298204; doi:10.3390/children13060774)
Supplement: Supplementary file 1 [file children-13-00774-s001.zip › children-4064248-supplementary.pdf]

# Outpatient Therapies

Please complete the survey below.

Thank you!

Participant ID ____________________

What therapies is your child currently receiving?
☐ Physical Therapy
☐ Occupational Therapy
☐ Speech Therapy
☐ Feeding Therapy
☐ Other
☐ None

## Physical Therapy

Physical Therapy
◯ Yes
◯ No

Are you receiving Physical Therapy weekly or monthly?
◯ Weekly
◯ Monthly

How many times a week is your child receiving therapy? ____________________

How many times a month is your child receiving therapy? ____________________

Is Physical Therapy treatment still ongoing?
◯ Yes
◯ No

Physical Therapy Start Date ____________________

When did Physical Therapy end? ____________________

## Occupational Therapy

Occupational Therapy
◯ Yes
◯ No

Are you receiving Physical Therapy weekly or monthly?
◯ Weekly
◯ Monthly

How many times a week is your child receiving therapy? ____________________

How many times a month is your child receiving therapy? ____________________

Occupational Therapy Start Date ____________________

| | |
|---|---|
| Is occupational therapy treatment still ongoing? | ◯ Yes<br>◯ No |
| When did occupational therapy end? | __________________________________ |

## Speech Therapy

| | |
|---|---|
| Speech Therapy | ◯ Yes<br>◯ No |
| Are you receiving Speech Therapy weekly or monthly? | ◯ Weekly<br>◯ Monthly |
| How many times a week is your child receiving therapy? | __________________________________ |
| How many times a month is your child receiving therapy? | __________________________________ |
| Speech Therapy Start Date | __________________________________ |
| Is speech therapy treatment still ongoing? | ◯ Yes<br>◯ No |
| When did speech therapy end? | __________________________________ |

## Feeding Therapy

| | |
|---|---|
| Feeding Therapy | ◯ Yes<br>◯ No |
| Are you receiving Feeding Therapy weekly or monthly? | ◯ Weekly<br>◯ Monthly |
| How many times a week is your child receiving therapy? | __________________________________ |
| How many times a month is your child receiving therapy? | __________________________________ |
| Feeding Therapy Start Date | __________________________________ |
| Is feeding therapy treatment still ongoing? | ◯ Yes<br>◯ No |
| When did feeding therapy end? | __________________________________ |

## Other 1

| | |
|---|---|
| Other Therapy Name (1) | ________________________ |
| Are you receiving Other Therapy weekly or monthly? | ◯ Weekly<br>◯ Monthly |
| How many times a week is your child receiving therapy? | ________________________ |
| How many times a month is your child receiving therapy? | ________________________ |
| Other Therapy (1) Start Date | ________________________ |
| Is other therapy (1) treatment still ongoing? | ◯ Yes<br>◯ No |
| When did other therapy (1) end? | ________________________ |
| Are there other therapies you go to that are not listed above? | ◯ Yes<br>◯ No |

## Other (2)

| | |
|---|---|
| Other Therapy Name (2) | ________________________ |
| Are you receiving Other Therapy (2) weekly or monthly? | ◯ Weekly<br>◯ Monthly |
| How many times a week is your child receiving therapy? | ________________________ |
| How many times a month is your child receiving therapy? | ________________________ |
| Other Therapy (2) Start Date | ________________________ |
| Is other therapy (2) treatment still ongoing? | ◯ Yes<br>◯ No |
| When did other therapy (2) end? | ________________________ |

## Therapy Name

| | 0 | 1 time per week | 2 times per week | 3 times per week | >3 a week | Once a month | Twice a month | Thrice a month |
|---|---|---|---|---|---|---|---|---|
| Physical therapy | ○ | ○ | ○ | ○ | ○ | ○ | ○ | ○ |
| Occupational therapy | ○ | ○ | ○ | ○ | ○ | ○ | ○ | ○ |
| Speech therapy | ○ | ○ | ○ | ○ | ○ | ○ | ○ | ○ |
| Feeding therapy | ○ | ○ | ○ | ○ | ○ | ○ | ○ | ○ |
| Other therapy 1 | ○ | ○ | ○ | ○ | ○ | ○ | ○ | ○ |
| Other therapy 2 | ○ | ○ | ○ | ○ | ○ | ○ | ○ | ○ |
